# “I always prefer to withdraw than use a condom”: contextualising condomless sex among East Asian and sub-Saharan African international students in Sydney

**DOI:** 10.1186/s13690-021-00777-z

**Published:** 2022-01-05

**Authors:** Sylvester R. Okeke

**Affiliations:** Centre for Social Research in Health, UNSW, Sydney, Australia

**Keywords:** Blood-borne viruses, STIs, HIV, Pleasure-seeking, Alcohol use, Stigma, Gender

## Abstract

**Background:**

Incidence and prevalence of blood-borne viruses and sexually transmissible infections among young people continue to necessitate population-based studies to understand how contextualised sexual health services can be developed and implemented to promote protective behaviours such as consistent condom use. This study examined condomless sexual practice among a sample of East Asian and sub-Saharan African international university students in Sydney, Australia.

**Methods:**

This qualitative study was methodologically guided by interpretative phenomenological analysis. Data was provided by 20 international students sampled from five universities in Sydney, who participated in either face-to-face or telephone semi-structured in-depth interviews. The interview sessions were audio-recorded, transcribed verbatim, coded in NVivo and analysed using reflexive thematic analysis.

**Results:**

Condomless sexual practices appear to be common among the study group based on participants’ self-reports of their own practices and the practices of friends and peers. Three themes contextualising condomless among the study participants were generated from the interview transcripts: (1) unanticipated sex, condom related stigma and alcohol use (2) pleasure-seeking, curiosity and intimacy (3) condomless sex as a gendered practice.

**Conclusions:**

The result of this study has implications for public health research, practice and policy around design, implementation and evaluation of multi-layered and population-specific sexual health services that are tailored to addressing the needs of international students, who  migrate from traditional sexual cultures to Australia, where sexual norms are more liberal.

## Background

Despite progress in improving sexual and reproductive health outcomes of young people in recent decades, they continue to constitute a key population for transmitting blood-borne viruses (BBVs) and sexually transmissible infections (STIs). With less than a decade to 2030—the target date set by international agencies to end the epidemics of HIV/AIDS, STIs and viral hepatitis [1, 2]—the number of global new cases of HIV among young people remains one of the obstacles to meeting this goal [3]. Compared to the general population, young people in Australia are at higher risks of STIs, BBVs and unplanned pregnancies [4]. While condom use is protective against STIs and some BBVs such as HIV and HBV [5], its correct and consistent use among young people, despite availability, seems to be a major challenge. In Australia, condoms are available in supermarkets/pharmacies for a price and at sexual health clinics for free.

Globally, condom use among young people is still low, despite research indicating that they possess relatively high level of knowledge about its efficacy in preventing STIs/BBVs and unplanned pregnancies [6–8]. Therefore, condomless sex has been identified as a common cause of STI and BBV infection among young people [9]. A substantial amount of research has been undertaken to assess condom use and its determinants among young people in a variety of settings [6, 10–12]. The present study adds to this body of knowledge by contextualising condomless sex among East Asian and sub-Saharan African international students in Sydney, based on their lived experiences and construction of their social realities.

The need to contextualise and understand condomless sex norms and practices among international students in Australia is important for two reasons. First, adolescents and young people do not constitute a homogenous group [13]. Most of what is known about condomless sex in Australia may not reflect the realities of international students, particularly as studies that have examined condom use behaviour among young people in this setting are disproportionately focused on Australian-born young people [6, 14–16]. Therefore, interventions based on these studies may not consider the range of different needs of international students. Australia continues to record a steady rise in the number of international students, and many come from settings where BBV/STI prevalence is high and where youth sexual health programmes may be inadequate or non-existent [17, 18].  Thus, there is a need to provide sexual health programmes that are tailored to the specific needs of this population. Such programmes will be impactful if they are evidence-based and evidence around sexual health of international students in Australia is still evolving [19–23]. The present study adds to this emerging body of evidence.

Second, this study aims to develop a better and more nuanced understanding of condomless sex experiences based on the lived experiences and perspectives of participants. Qualitative research offers rich and compelling insights into participants perspectives and experiences in ways that may be entirely different from quantitative research [24]. Despite the usefulness of qualitative research in understanding and contextualising health behaviour, most of what we know about condom use among young people in Australia is based on quantitative research [6, 14–16]. Though quantitative studies provide an account of condom use trends/patterns and associated factors; they do not enable a detailed understanding of individual or group experiences.

Consequently, research providing a more nuanced and in-depth understanding of the sexual experiences of young international students is important to inform effective interventions that address population-specific and multi-layered factors that may be intersecting to shape and reinforce condomless sex among this population. Using a qualitative design, the present study moves beyond examining trends, patterns and associations and explores the experiential and social dimensions of condomless sexual practice as well as descriptive and injunctive norms around this practice among a sample of international students in Sydney, Australia. Injunctive and descriptive norms as used in this study refer to what participants perceive as *expected* behaviour (injunctive) against *actual* or *normal* behaviour (descriptive) [25] as it relates to condom use in sexual encounters.

## Methods

### Study design and paradigm

This qualitative study is part of a larger mixed methods research project that investigated STIs/BBVs risk and protective practices among East Asian and sub-Saharan African international university students in Sydney. Theoretically, it adopts an interpretivist paradigm, methodologically guided by Interpretative Phenomenological Analysis (IPA) and reported using relevant items from the Consolidated Criteria for Reporting Qualitative Research (COREQ) checklist [26]. Phenomenology as originally developed by Edmund Husserl (1859–1938), now known as descriptive or transcendental phenomenology, involves describing the experiences of research participants outside the perceptions or belief system of the researcher [27]. Meanwhile, the approach to phenomenology used in this study is the interpretive or hermeneutic phenomenology developed by Martin Heidegger (1889–1976), which considers a researcher’s belief system as central to interpreting the world around and making meaning from the lived experiences of study participants [27]. *Experience* in phenomenology, as applied in this study, also involves participants’ perception, imagination, thought, memory and emotions around condoms [28].

Furthermore, in hermeneutic phenomenology, historical context is critical to understanding any experiences being explored [27]. As such, building this study around this paradigm enabled a more nuanced understanding of the complexities surrounding how participants’ sexual and condom-related practices in Sydney are shaped by their past experiences, especially back in their home backgrounds [29]. Moreover, the researcher’s insider positionality, interest and presuppositions transparently shaped the aims, questions and knowledge claims of this study [30, 31] and serve as resources, rather than prejudices that could affect the study results [32].

### Sample and recruitment

Data was provided by 20 participants—10 each from East Asia (6 females and 4 males) and sub-Saharan Africa (5 females and 5 males)—through semi-structured in-depth interviews, which were conducted between May and August 2019. Individual interviews provided an environment where participants may be more comfortable to discuss their experiences; considering that  sexual issues are treated discreetly by young people from conservative cultural backgrounds [21, 33]. Inclusion criteria included being an international university student from East Asian or sub-Saharan African country, currently studying on a student visa in undergraduate or postgraduate courses, being 18 years and above and having lived in Sydney for at least three months. Importantly, all domestic students, including Australian permanent residents or citizens of East Asian or sub-Sharan African backgrounds, were not eligible to take part in this study. This is because as domestic students, they may have different experiences compared to international students, whose temporary visa status may predispose to some structural vulnerabilities that domestic students of the same backgrounds may not experience. Moreover, while sexual interactions between international and local students are possible, the scope of the present study does not include exploring the condom use experiences of local students or comparing their experiences with those of international students. Therefore, domestic students, including those from African or Asian backgrounds were excluded from the study.

Recruitment was done through a combination of pragmatic strategies involving electronic and paper flyers, which were used to advertise the study at various international students’ social media platforms as well as on strategic locations at university campuses. The study also used snowballing sampling strategy which involved participants sharing the recruitment flyers and inviting other international students within their networks to participate [34]. However, only one participant was successfully recruited through snowballing. Interested participants contacted the researcher through email or by phone to arrange interviews. Ethical approval for the study (HC190215) was provided by the University of New South Wales (UNSW) Human Research Ethics Committee. All the study participants provided informed consent either in writing (face-to-face) or verbally (telephone) before all the interviews. Consent also involved participants’ understanding that participation was voluntary, that they could withdraw at any time and that findings will be published in academic journals.

### Data collection procedure

Since this study was built on IPA, its overarching aim was to examine how international students, as sense-making creatures, make sense of condomless sex-related experiences in Australia [35]. As a participant-oriented approach, which requires participants to tell their own stories, from their own perspectives and in their own words [35, 36], semi-structured interviews were deemed most appropriate methods of data collection as these interviews enabled the flexibility to reflexively accommodate, probe and explore emerging issues during the interviews. This allowed greater engagement and bonding with the participants in a conversational and an interactive manner. Aside responding to the interview questions, this interview approach enabled participants to speak about other contexts and perspectives  that they feel are also important to the overall focus of the study [37].

At the point of arranging for the interviews, participants were asked if they would prefer to be interviewed by someone of the same gender and/or sub-region. Since none of the participants indicated preferences for an interviewer, all the interviews were conducted by the researcher, who is from sub-Saharan Africa and identifies as a male and a heterosexual. Participants were interviewed about their sexual practices in Australia and their condom use experiences as well as those of their friends and other international students from the same sub-region. Participants received a $40 gift voucher as reimbursement for costs that may be associated with participating in the interviews. All the interviews were conducted in English, and they lasted between 40 and 85 min.

### Data analysis

The interviews were audio-recorded and transcribed verbatim for analysis. Considering that the study’s methodology is built on IPA, participants experiences were illustrated and reported thematically [27]. In using thematic analysis, the study adopted the reflexive thematic analysis (RTA) which emphasizes the importance of researchers’ subjectivity as analytic resource [30, 32] and fits the fundamental flexibility assumptions of IPA [27]. The theoretical orientation guiding the way in which meaning was made from the data during analysis is that interview data and field notes represent both experiential and social discourses that perform social actions [30]. Therefore, theoretically, RTA, as used in this study, combined elements of phenomenology and critical qualitative research.

The researcher iteratively read through the data transcripts and field notes while listening to the interviews, multiple times. This was done for familiarisation, immersion and understanding of the meaning conveyed in the data beyond its semantic representation [30]. The transcripts were thereafter coded using QSR NVivo (12.6.0). Coding combined inductive and deductive strategies [30], as IPA—though inductive and grounded in generated data—also acknowledges dominant literature [27]. However, generated themes are not domain summaries [38] but identified patterns of shared meaning in the study data [32]. While reviewing, refining and naming initial themes, the researcher kept checking back through the data to ensure that they adequately reflect participants’ accounts. A more detailed description of the study methods has been published elsewhere [21].

## Results

### Sociodemographic characteristics of participants

Most participants, eight out of twenty, were aged between 18 and 24 years. Nineteen of the twenty participants identified as heterosexuals while the remaining participant identified as gay. Similarly, most participants, sixteen out of twenty were never married and eleven of the twenty participants identified as females. Further, thirteen of the twenty participants reported that they are sexually active in Australia (Table 1).

**Table 1:** Socio-demographic characteristics of participants

Variable	Frequency (%)
***Sex***	
Female	11 (55.0%)
Male	9 (45.0%)
***Age***	
18–24 years	8 (40.0%)
25–29 years	6 (30.0%)
30–39 years	6 (30.0%)
***Marital status***	
Single	16 (80.0%)
Married	4 (20.0%)
***Background***	
East Asia	10 (50.0%)
Sub-Saharan Africa	10 (50.0%)
***Sexual orientation***	
Heterosexual	19 (95.0%)
LGBTQI+	1 (5.0%)
***Sexually active in Australia***	
Yes	13 (65.0%)
No	7 (35.0%)

### Themes contextualising condomless sex

Patterns in the data showed themes describing contexts were condomless sex occurs as well as injunctive and descriptive norms around this sexual practice. These themes are grouped into three: [1] unanticipated sex, condom related stigma and alcohol use [2] pleasure-seeking, curiosity and intimacy [3] condomless sex as a gendered practice.

#### Unanticipated sex, condom related stigma and alcohol use

The results indicate that condomless sex occurs within the context of unanticipated sexual encounters. Some participants who reported recent condomless sex disclosed that they were not in possession of a condom at the time. Some of them also mentioned that these sexual encounters were not anticipated.

*It [sex] was impromptu, it [condom] was not available* (female/31/sub-Saharan Africa).

Interview data suggests that participants understand the risks associated with unanticipated sex and how carrying condoms around could help in preventing these risks. In fact, one of the participants used metaphors to emphasize a need for young people to always carry condoms.

*[it is] a raincoat (condom). You carry it around because you don’t know when it will rain (unanticipated sex). If you don’t have it and the rain starts, you [will] just get wet (get infected with BBV/STI)* (male/30/sub-Saharan Africa).

Further, although it could be argued that young people do not have condoms handy because they may not know where to get them or that purchasing condoms may be expensive, the interviews indicate that this may not be the case for these group of students as some reported that they are aware of settings where they can access free condoms:

*… when I went to the medical centre, I actually saw a place, a stand where they placed free condoms for people, if they want to have sex* (male/19/sub-Saharan Africa).

*… it [condomless sex] is sad, because condom is not expensive. [Condom] availability is not a problem, it is always there, anyone can acquire and can afford it as well* (male/29/sub-Saharan Africa).

However, one participant noted that freely distributed condoms are of low quality and interfere with sexual pleasure and intimacy.

*Free condoms are really bad quality. The free condom is like more rubber … I tried the free condoms from the university public health service. That is really bad. I will not use this condom [again]. … If you really want to have [unhindered pleasurable sex], you need to spend your money to buy the good brand condoms. That might be better* (female/36/East Asia).

Thus, generated data indicate that though condoms may be freely available and affordable, there may be other reasons that these young people do not have condoms on them. The interviews suggest that they may feel uncomfortable and reluctant to carry condoms around for fear of being judged because of premarital abstinence expectations. A participant reported that participating in the interview had caused him to reflect on his views that carrying a condom around was disrespectable:

*Maybe before now [before participating in the interview], I will say it wasn’t so nice, it wasn’t so presentable for a person, but you can’t really judge a person for carrying condom* (male/29/sub-Saharan Africa).

Since participants come from more traditional cultures with conservative sexual norms [21]; condoms may be highly stigmatised in these settings and associated with promiscuous sexual activities [39, 40]. Coming from a conservative culture may potentially affect how young people from these settings feel about accessing and/or carrying condoms around in Australia.

*… let me tell you why. The culture in [home country] is so disgusting. They give you this stigma, they give this kind of eye like [participant demonstrates such disdainful facial expression] … That’s why you find it very hard to see [young] people going to buy it [condom]. I have some people, then, that they find it very hard to go and get it [condom]. ... if they see it [condom] with you, the facial expression they will give you … even the person selling it will give you [disproving] facial expression* (female/29/sub-Saharan Africa).

In contrast to the more conservative norms in their home country, some participants feel that there is little or no stigma around accessing, purchasing or being in possession of condoms in Australia.

*… they are not really comfortable going and request [ing] for it [condom], … but over here, I don’t see it [accessing condoms] as a problem. People walk into pharmacies and shops and they buy and because the awareness is really effective over here, people don’t really find it to be a problem purchasing [condoms]* (male/29/sub-Saharan Africa).

Interestingly, findings also indicate that even in Australia, accessing and carrying condoms around may not translate to actual use, even in planned sexual encounters. The interviews suggest that sociocultural norms stigmatise condoms by associating being in possession of condoms with sexual permissiveness and this may intersect with gender stereotype to encourage condomless sex:

*But can I tell you one thing, basically, in respect of women, they don’t really say it, but we feel it as women. If you come to someone’s place ready, like having condoms in your bag, it doesn’t give you a good impression, you don’t come off as a decent woman because they expect a woman not to be expecting sex; even if in such a case when we have an online chat and I am going to someone’s place for that reason [to have sex], it doesn’t look right in his eyes to see [me] bringing condoms. They look at women in a different way and I have shared this idea with my other friends, they say “sometimes we have it [condom]; but we are too embarrassed to say we came prepared.” That will indicate she is used to having so much sex, that way, she doesn’t look great in his eyes* (female/23/East Asia).

An additional reason reported by some participants for not using condoms is when sex occurs under the influence of alcohol. Participants reported that sex under the influence of alcohol weakens personal decisions not to engage in condomless sex.

*For me, nothing would make me want to have sex without a condom, unless I’m drunk. That’s it. [Probe: Okay. Have you ever been?]. Yeah, that was the time that we didn’t use a condom. [It] was when I was drunk. The person that I had unprotected sex with wasn’t the guy I’m seeing [dating]. It was another guy* (female/19/sub-Saharan Africa).

#### Pleasure-seeking, curiosity and condom use fatigue

The interviews showed that condom use among participants is complex, and not just related to condom availability. A participant described a gap between having a condom and remembering to use it.

*… but it’s always not easy ... [you] do not even think of that [using condom], so, it’s always really hard to remember … even if you have the condom in the pocket … Most of the time it’s hard. For me, I always prefer to withdraw than use a condom* (male/27/sub-Saharan Africa).

Moreover, pleasure-seeking is also cited as a reason for condomless sex occurrence based on personal accounts and description of social practice among members of the study population. The need to increase pleasurable experience could be contributing to condomless sex among the group as condoms are perceived as barriers to sexual pleasure.

*So, based on my experience, I think, one thing could be like … want [ing] to find more pleasure, want [ing] to find more exciting experience* (male/22/East Asia).

*I think people just love the pleasure that comes without using a condom … they feel more satisfied, more connected with whatever individual they are having a sexual experience with without a condom. So, I think that is why, basically, they tend to go without it* (male/29/sub-Saharan Africa).

Not using a condom may also occur out of a desire for deeper connection and intimacy with a partner. Aside inhibiting sexual pleasure, condoms may also be viewed as barrier to intimacy in sexual interactions.

*Because many people complain that they have some different feelings. They think that condom materials are uncomfortable or it’s isolating them from each other … condoms should have some other materials that improve the quality of the condom. So, when people use the condom, they don’t feel different* (female/36/East Asia).

Further, curiosity was equally reported as a reason for engaging in condomless sex. Some participants narrated experiences where they engaged in condomless sex for the purpose of experimentation and satisfying their curiosity around what condomless sex feels like.

*I think it was … maybe the curiosity* (female/22/sub-Saharan Africa).

*First time I think I just wanted to try it [condomless sex]. I just tried it for a while and then I used condom … to make it safe* (male/22/East Asia).

Condomless sex may also be linked to condom use fatigue over time as sex with the same partner may become condomless with time. Results indicate that this sporadic use of condoms may be attributed both to condom use fatigue and feelings of ‘trusting’ a partner with time. The occurrence of condomless sex in this context was reported among male and female participants.

*At some point, if you’re in a relationship with someone and you’ve been using condoms … you get to a point where there’s this sort of trust that comes and you decide not to use condoms. … So, that’s kind of the point where I will chuck away the use of condoms. … It has happened with more than three people* (male/32/sub-Saharan Africa).

*there would always be a point … where you wouldn’t use protection, … especially with someone that you’re dating. So, there were times when we didn’t use stuff [condom] like that* (female/29/sub-Saharan Africa).

#### Condomless sex as gendered practice

The interview data indicate that condomless sex practices and norms appear to be gendered and this includes the way condoms are accessed. Women reported that they rely on men to provide condoms and condomless sex may likely occur when this expectation is not met.

*I like being protected but I rely on the guy to have condoms and [at] those certain times, there was none … we just had sex without condoms* (female/23/East Asia).

Results also indicate that participants associate condomless sex with gender negotiations and power. Although this  study's results in this regard are complex, women and girls are perceived by both male and female participants to be gatekeepers, who have the power to decide whether sex will be condomless or protected.

*… and then maybe the men don’t like it [condom]. I don’t know why. … for me, if [a] man wants to have sex with me and doesn’t listen to me [to use a condom], then I will ignore him. I just leave him, but I don’t know why other girls just don’t leave?* (female/36/East Asia)

This experience is also echoed by a younger participant:

*I know some guys; they probably don’t want to use [condoms] and the girls just allow it* (female/24/East Asia).

The gendered view relating to condomless sex was also linked to a socio-cultural norm that associates condom use with female promiscuity or sex work in conservative cultures:

*Like in my culture, having sex with a lady using protection [condom] can be very difficult because … [that means that] you have defined her to be a prostitute … “It means you don’t trust me, and so you feel I’m going around having sex with so many people.” So, that belief is there* (male/30/sub-Saharan Africa).

## Discussion

 Results suggest that though participants are aware that condomless sex could increase the risk of contracting BBV/STI—as well as where to access condoms—they still practice condomless sex due to a range of reasons. Notably, the fear of feeling judged, plays a role in condomless sex among some participants, as it affects condom access as well as its use, even when it is available. This stigma may be attributed to socio-cultural expectation about premarital sexual abstinence [12] and norms associating condoms with infidelity and sexual permissiveness [10, 39, 40]. Despite migrating to a setting with a more liberal sexual culture, sexual perceptions and practices of young people from conservative cultures may still be partly influenced by their home cultural sexual norms [21]. Therefore, stigmatising socio-cultural norms affecting condom access and use in the conservative sexual cultures of participants’ backgrounds [39, 41], may still affect their condom use practices in Sydney. Although condom availability increases likelihood of use [42], results of the present study indicate that reluctance to use condoms for several reasons, including condom-related stigma, could discourage some young people from accessing and being in possession of condoms. This result is also supported by findings of previous studies among participants in settings where conservative sexual cultures are prevalent [11, 12, 43]. Consequently, sexual health interventions designed for this group of international students should address lack of confidence in accessing condoms as well as reluctance to use a condom when it is available.

Additionally, social stigma associating condoms with sexual permissiveness may also intersect with gender stereotypes to promote condomless sex. This is however complex as some female participants reported sexual agency in sexual negotiations by insisting that their male partners use a condom. On the other hand, some female participants seem to be influenced by cultural norms that associate condoms with sexual permissiveness. Similarly, while some female participants access condoms, others rely on their male partners to provide the condoms. Interestingly, this study's results showed that male and female participants described women as gatekeepers for condom use in sexual relationships, as they believe that women have the power to make decisions about condom use.

Sexual agency demonstrated by some female participants in negotiating condom use—in line with previous studies [44, 45]—contradicts traditional stereotypes that portray East Asian and sub-Saharan African women as compliant and lacking power to negotiate condom use [11, 46, 47]. However, considering that some female participants may still feel less confident to access and negotiate condom use, it may be necessary for interventions to focus on improving sexual assertiveness and negotiation skills of international students to boost their confidence in accessing, negotiating and using condoms as low condom negotiation among women in particular increases likelihood of condomless sex [48]. Empowering women’s ability to assertively negotiate condom use is crucial as unsafe sex is a leading cause of infection among women globally [49].

Aside condom related stigma and gendered negotiations, the study results also showed that despite some participants reporting being aware of the risks inherent in condomless sex, the need to satisfy curiosity around what it feels like to have sex without a condom, compels them to ignore these risks. Previous studies have also linked curiosity to other sex related health-risk practices such as unprotected multiple sexual relationships [50] and unprotected oral sex [51]. Similarly, the pleasure-seeking–characterised by ignoring risks associated with condomless sex in order to attain maximal pleasure–may also need to be addressed in sexual health interventions for the population as ignoring the need to stay safe to opt for pleasure-seeking was found to be a reason why some participants engaged in a recent condomless sex. This finding aligns with results of previous studies that reported pleasure-seeking as a factor in condomless sexual practices among young people in East Asian [11] and sub-Saharan African [7] settings. Addressing pleasure-seeking occurring within the context of ignoring risks may require a dual effort that combines behavioural interventions [52–56] along with addressing young people concerns about condoms inhibiting sexual pleasure. To increase acceptability and use, condoms should be more sensitive. Similarly, condom promotion messages should also include sensitisations about lubricants that enhance sensitivity and increase pleasure.

Furthermore, the implication of alcohol use in condomless sex as reported in previous studies [45, 56, 57] also resonates in the present study. Against this backdrop, the population of the present study could benefit from sexual health programs that embed socio-decision making and self-regulation skills, which have been found to be protective against risky sexual practices [58, 59] as well as unhealthy drinking and condomless sex [60–62].

The results of this study need to be understood within some caveats. The study appears to be heteronormative in approach and analysis as heterosexuals accounted for 95% of the study participants. Although intensive efforts were made to recruit participants from LGBTQI+ community groups, the researcher was cautious of coercion. This is important, considering that international students from conservative cultures, who are not heterosexuals, may feel awkward to openly identify as non-heterosexuals because of their cultural socialisation. Therefore, this limitation may impact on the relevance of this study's results to international students, who are not heterosexuals and to some extent, bisexuals.

## Conclusion

Contexts for condomless sex among the study participants are multiple and this has implications for multi-layered and tailored sexual health services that address intersecting barriers against consistent condom use. This would ensure that international students are better empowered to adopt safer sexual practices and consciously protect themselves against BBVs, STIs and other consequences of unsafe sexual practices. Although condomless sex may not equate with unsafe and high-risk practices as other approaches such as PEP, PrEP and vaccinations could also provide protection, the study population seems to have higher levels of awareness, access and acceptance of condoms, compared to these alternatives. Therefore, improving condom use practices among the population, while creating awareness and access to these other alternatives, will ensure the provision of multi-faceted interventions needed to address the complex and diverse sexual health needs of the study population, just as other young people.

## Data Availability

De-identified data is available on reasonable request.

## Abbreviations

AIDS: Acquired immunodeficiency syndrome
BBVs: Blood-borne viruses
COREQ: Consolidated criteria for reporting qualitative research
HBV: Hepatitis B virus
HIV: Human immunodeficiency virus
IPA: Interpretative phenomenological analysis
LGBTQI: Lesbian, gay, bisexual, transgender, queer and intersex
PEP: Post-exposure prophylaxis
PrEP: Pre-exposure prophylaxis
RTA: Reflexive thematic analysis
STIs: Sexually transmissible infections
